# Supplementary material for: ZnO-Loaded Graphene for NO2 Gas Sensing
Source: Sensors (Basel). 2023 Jun 30;23(13):6055. doi: 10.3390/s23136055 (PMC10346611; doi:10.3390/s23136055)
Supplement: Supplementary file 1 [file sensors-23-06055-s001.zip › sensors-2296539-supplementary.pdf]

Support information for

ZnO loaded graphene for NO_2 gas sensing.

M.A. Alouani, J. Casanova-Chafer, F. Güell, E. Peña-Martin, S. Ruiz Martinez-Alcocer, S. de Bernardi-Martin, A.García Gómez, X. Vilanova* and E. Llobet

EDS for 95-5 sensor:

A qualitative and semi-quantitative analysis performed on the surface of the 95-5 sensor via the energy dispersive spectroscopy (EDS) technique, the results obtained shown in the figures below showing mappings of the elements present on the surface of the sensitive layer with different colors proving the presence of the ZnO nanoparticles. Quantitatively, carbon, oxygen and aluminum have the strongest peaks since carbon is coming from the graphene layer, oxygen is abundant on the surface of the studied area, and aluminum coming from the alumina substrates which the layers were deposited on top. Zinc element has a weak peak since its concentration on the surface is too low especially in a small chosen area but still it was proven to be present with an atomic % of 0.53%, meanwhile the Si peak which is almost hard to see comes also from the alumina substrate.

(a)

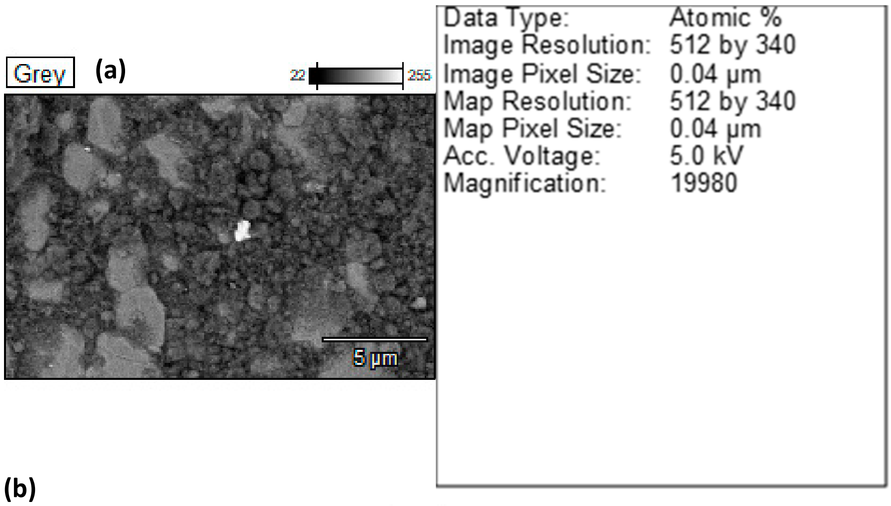

Data Type:	Atomic %
Image Resolution:	512 by 340
Image Pixel Size:	0.04 µm
Map Resolution:	512 by 340
Map Pixel Size:	0.04 µm
Acc. Voltage:	5.0 kV
Magnification:	19980

(b)

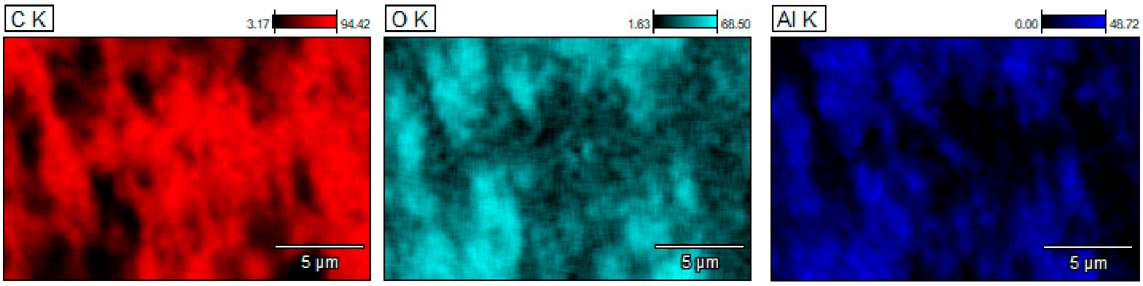

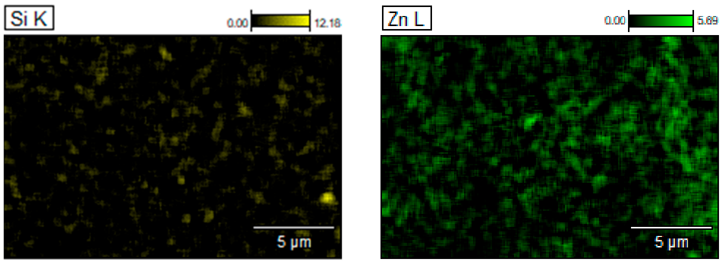

(C)

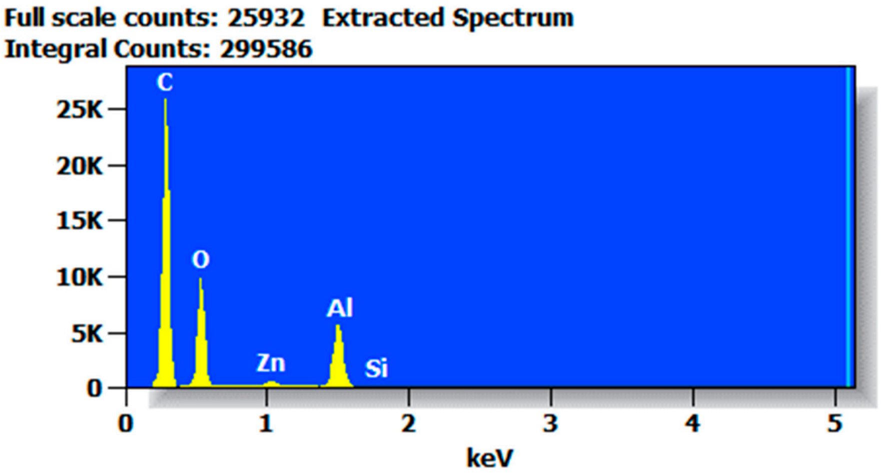

Figure S1. (a) Image of the chosen area of G95-5 sensitive layer for the mapping taken by FESEM (b) mapping images of the elements present in the studied area (c) the extracted spectrum from the EDS analysis.

Table S1. Characteristics of the elements present in the studied sensitive layer.

**Element**	Weight %	Weight % err	Atom %	Norm. Wt. %	Chemical Formula
**C K**	37.70	0.17	50.33	37.70	C
**O K**	32.91	0.21	32.98	32.91	O
**Al K**	26.61	0.17	15.81	26.61	Al
**Si K**	0.62	0.10	0.35	0.62	Si
**Zn L**	2.17	0.12	0.53	2.17	Zn
	100.00		100.00	100.00	

EDS for 80-20 sensor:

Same EDS analysis was performed on the surface of the 80-20 sensor, and as expected the intensity of the peaks are different since certain elements are more abundant such as oxygen and zinc since the concentration of the ZnO NPs on the surface of the graphene is 20%, this explains the EDX spectrum with oxygen (O) having the strongest peak, aluminium (Al) peak is weaker since the substrate is more covered with the material and then comes carbon coming from the graphene. Zinc also shows stronger peak than the one in the previous EDS analysis of the 95-5 sensor since its more present on the surface of the substrate with an atomic % of 4.03%, magnesium (Mg) and silica (Si) peaks are almost invisible since they come from the alumina substrate with very low weight %.

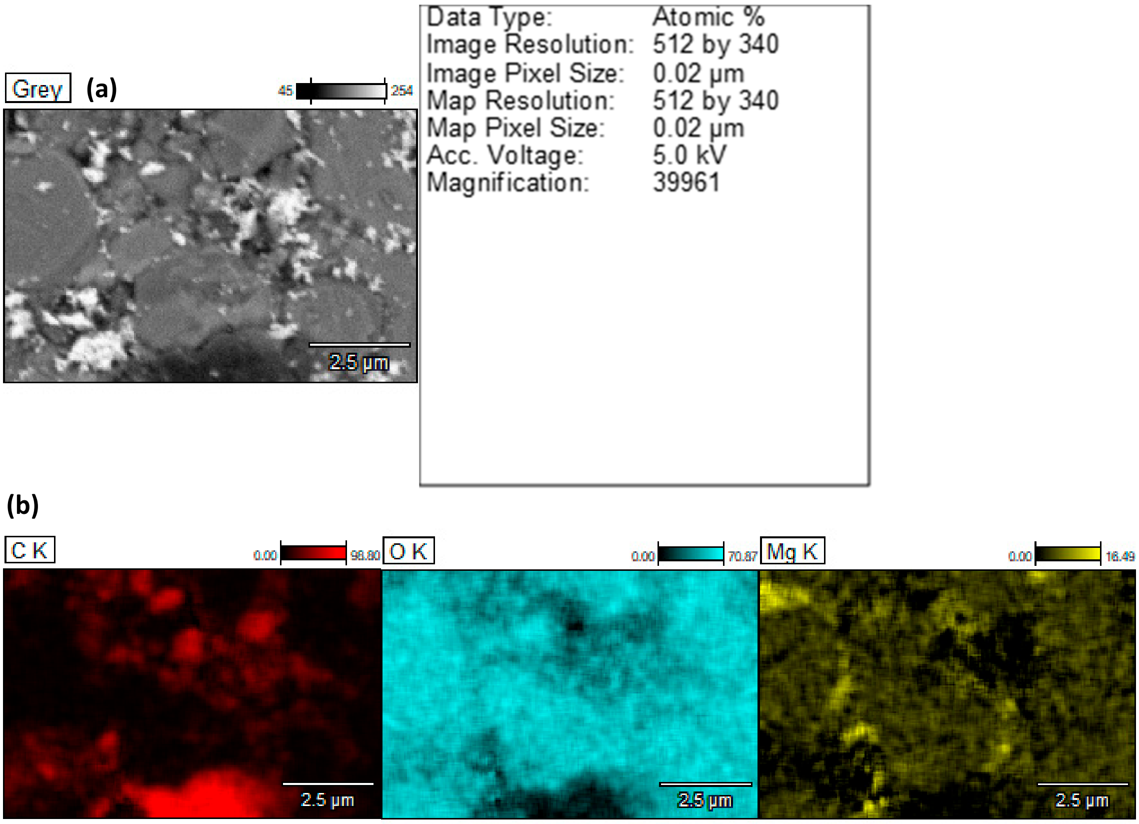

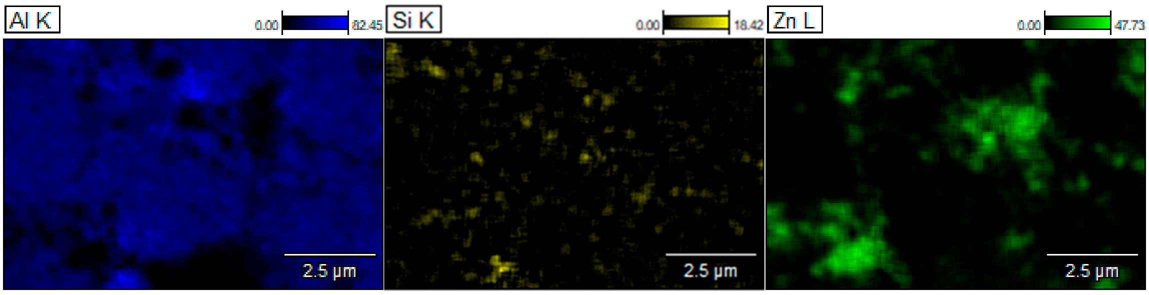

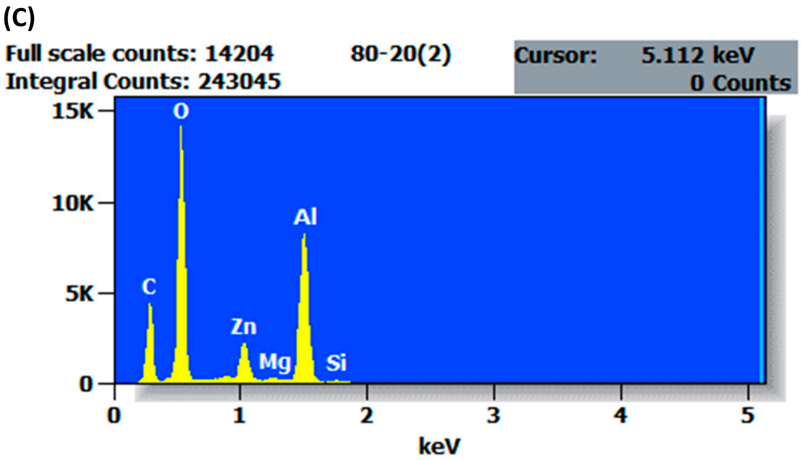

Figure S2. (a) Image of the chosen area of G80-20 sensitive layer for the mapping taken by FESEM (b) mapping images of the elements present in the studied area (c) the extracted spectrum from the EDS analysis.

Table S2. Characteristics of the elements present in the studied sensitive layer.

**Element**	Weight %	Weight % err	Atom %	Norm. Wt. %	Chemical Formula
**C K**	8.44	0.05	14.60	8.44	C
**O K**	38.99	0.22	50.64	38.99	O
**Mg K**	0.44	0.05	0.38	0.44	Mg
**Al K**	38.91	0.21	29.96	38.91	Al
**Si K**	0.53	0.05	0.40	0.53	Si
**Zn L**	12.68	0.16	4.03	12.68	Zn
	100.00		100.00	100.00	
